# Engineering Red-Enhanced and Biocompatible Upconversion Nanoparticles

**DOI:** 10.3390/nano11020284

**Published:** 2021-01-22

**Authors:** Masfer Alkahtani, Najla Alsofyani, Anfal Alfahd, Anas A. Almuqhim, Fahad A. Almughem, Abdullah A. Alshehri, Hussam Qasem, Philip R. Hemmer

**Affiliations:** 1 King Abdulaziz City for Science and Technology (KACST), Riyadh 11442, Saudi Arabia; nalsofyani@kacst.edu.sa (N.A.); aalfahd@kacst.edu.sa (A.A.); amukhem@kacst.edu.sa (A.A.A.); hqasem@kacst.edu.sa (H.Q.); 2 Institute for Quantum Science and Engineering, Texas A&M University, College Station, TX 77843, USA; prhemmer@exchange.tamu.edu; 3 National Center for Pharmaceutical Technology, King Abdulaziz City for Science and Technology (KACST), Riyadh 11442, Saudi Arabia; falmughem@kacst.edu.sa (F.A.A.); abdualshehri@kacst.edu.sa (A.A.A.); 4 Department of Electrical and Computer Engineering, Texas A&M University, College Station, TX 77843, USA; 5 FRC Kazan Scientific Center of RAS, Zavoisky Physical-Technical Institute, Sibirsky Tract, 10/7, 420029 Kazan, Russia

**Keywords:** upconversion nanoparticles, red-enhanced fluorescent markers, bioimaging, bioapplication

## Abstract

The exceptional optical properties of lanthanide-doped upconversion nanoparticles (UCNPs) make them among the best fluorescent markers for many promising bioapplications. To fully utilize the unique advantages of the UCNPs for bioapplications, recent significant efforts have been put into improving the brightness of small UCNPs crystals by optimizing dopant concentrations and utilizing the addition of inert shells to avoid surface quenching effects. In this work, we engineered bright and small size upconversion nanoparticles in a core–shell–shell (CSS) structure. The emission of the synthesized CSS UCNPs is enhanced in the biological transparency window under biocompatible excitation wavelength by optimizing dopant ion concentrations. We also investigated the biosafety of the synthesized CSS UCNP particles in living cell models to ensure bright and non-toxic fluorescent probes for promising bioapplications.

## 1. Introduction

Nanoscale luminescent materials have attracted a special interest in numerous biological applications including biological tissue imaging, super-resolution imaging, sensitive optical sensing, optogenetic (brain studies), and drug delivery. These include organic dyes [1,2,3], quantum dots (QDs) [4,5], and fluorescent polymers [6,7,8]; however, they are limited by either photostability [9], toxicity [10,11], or chemical environment sensitivity [12]. 

Fluorescent diamonds have been proposed to overcome the abovementioned drawbacks and shown great potential in many promising biological applications [13]. Among the major hurdles in the application of fluorescent diamonds is that the optical excitation of many interesting color centers, including the nitrogen-vacancy (NV) by blue and green wavelengths, has been reported to cause heating and photodamage to biological tissues even with relatively low power intensity, as low as 2 kW/cm^2^ [14]. Furthermore, the green excitation of the NV center induces autofluorescence in biological tissues resulting in a significant sensitivity reduction in deep tissue-imaging and biosensing [15,16]. While some of the diamond color centers can be excited and detected within the biological transparency [13,17], their optical properties often degrade as the size of the diamond crystal decreases to biologically advantageous sizes (less than 7 nm) [18]. 

To overcome the limitations of all these fluorescent markers, lanthanide ions doped upconversion nanoparticles (UCNPs) have been increasingly explored because of their ability to strongly suppress background biofluorescence by both spectral and time-gating filtering, owing to long luminescence lifetimes in the order of milliseconds [19,20,21]. A key property of UCNPs is that their excitation and/or fluorescent wavelengths can be easily tuned within the optical transparency window, where tissue scattering and absorption are minimum and photo-toxicity is greatly reduced [14]. To fully utilize the unique advantages of UCNPs for bioapplications, significant efforts have been put into improving the brightness of small UCNP crystals by the addition of optically inert shells to avoid surface quenching effects (such as nonradiative losses from surface defects and solvent ligands, etc.), which dominate due to the high surface-to-volume ratio [22,23,24,25]. Additionally, optimizing sensitizer and activator ion concentrations were proven to give the best brightness of UCNPs, enabling single-particle imaging at sub-10 Wcm^−2^ irradiance with a quantum yield reaching 5% [23]. 

These recent achievements in the UCNP field have opened the door to advancements in bright and non-toxic fluorescent markers for specific bioapplications. For example, the Neodymium (Nd^+3^) sensitizer has been used as an alternative to direct excitation of the most common UCNP ytterbium (Yb^+3^) sensitizer [26,27,28,29], because it shifts the excitation wavelength to about 800 nm. Here, the laser-induced local heating effect in living cells is minimized [26,30]. One drawback of this strategy is that an enhanced green emission of the erbium doped UCNPs under 800 nm excitation overlaps with the absorption band of the biological tissue absorption window, leading to an autofluorescence background [26].

To overcome this issue, here, we present a red-enhanced emission of 808 nm excited UCNPs using a core–shell–shell (CSS) structure, where sensitizer and activator ion concentrations are optimized to meet the requirements of biologically transparent fluorescent markers. We also investigated the toxicity of the synthesized particles on living cell models to evaluate the brightness and toxicity of our fluorescent probes for promising biosensing applications.

## 2. Materials and Methods

### 2.1. Lanthanide Stock Solutions

A 0.2 M lanthanide stock solutions were prepared by dissolving the particular lanthanide acetates in DI water. For a complete dissolution of lanthanide acetates in DI water, an amount of acetic acid equal to 2% of the weight of lanthanide acetates was added to the solution under ultrasonic treatment for approximately 15 min. The Lanthanide stock solutions were then stored in sealed containers at ~4 °C. 

### 2.2. NaOH-Methanol and NH_4_F-Methanol Stock Solutions 

Amounts of 1 M of NaOH and 0.4 M of NH_4_F were dissolved in methanol under ultrasonic treatment for approximately 15 min. The NaOH-methanol and NH4F-methanol stock solutions were stored in sealed plastic containers at ~4 °C for further use. 

### 2.3. Synthesis of NaGdF_4_:Yb/Er (20/8%) Core Nanocrystals

At room temperature, 1.44 mL of Gd(CH_3_CO_2_)_3_ stock solution, 0.4 mL of Yb(CH_3_CO_2_)_3_ stock solution, 0.16 mL of Er(CH_3_CO_2_)_3_ stock solution, 4 mL of oleic acid, and 6 mL of 1-octadecene were added into a 50 mL two-neck flask. The flask was left uncapped, and the resulting mixture was heated to 165 °C for 40 min under a moderate stirring speed to give a clear light-yellow solution. The mixture was then cooled naturally to room temperature under continuous stirring. Amounts of 1 mL of NaOH stock solution and 3.3 mL of NH4F stock solution were added into a 15 mL centrifuge tube and mixed by a vertex for 10 s and then quickly injected into the reaction flask. The resulting cloudy solution was heated to 50 °C and kept at this temperature for 40 min. Next, the solution was heated to 100 °C for 10 min to remove low-boiling solvent and then heated to 295 °C for 1 h under nitrogen atmosphere. After the reaction was complete, the reaction was stopped and cooled down slowly to room temperature under continuous stirring. After the solution was naturally cooled, 5 mL of ethanol was added, and the resulting mixture was centrifuged at 6000 rpm for 10 min to yield a compact pellet, and the supernatant was discarded. The produced particles were collected and washed with cyclohexane and ethanol (4 mL, 1:1, *v*/*v*) three times. Finally, the NaGdF4 core nanocrystals (50 mg·mL^−1^) were re-dispersed in 4 mL of a fresh cyclohexane and stored at ~4 °C.

### 2.4. Synthesis of NaGdF_4_:Yb/Er@NaYb_0.9_F_4_:Nd (10%) Core–Shell Nanocrystals

At room temperature, 1.8 mL of Yb(CH_3_CO_2_)_3_ stock solution, 0.2 mL of Nd(CH_3_CO_2_)_3_ stock solution, 4 mL of oleic acid, and 6 mL of 1-octadecene were added into a 50 mL two-neck flask. The flask was left uncapped, and the resulting mixture was heated to 165 °C for 40 min under moderate stirring to obtain a clear light-yellow solution. After that, the mixture solution was to cooled to room temperature under continuous stirring. Next, 3 mL of the prepared NaGdF4 core particles (37.5 mg mL^−1^), 1 mL of NaOH stock solution, and 3.3 mL of NH_4_F stock solution were injected into the reaction flask. The resulting cloudy solution was heated and maintained at 50 °C for 40 min. Next, the solution was heated to 100 °C for 10 min to remove low-boiling solvent and then heated to 295 °C for 1 h under nitrogen atmosphere. After the reaction was complete, the reaction was stopped and slowly cooled down to room temperature under continuous stirring. Finally, the subsequent purification steps are the same as those used for NaGdF4:Yb/Er (20/8%) core nanocrystals. The core–shell nanocrystals NaGdF4:Yb/Er@NaYbF4:Nd (10%) (86.4 m·mL^−1^) were dispersed in 4 mL of fresh cyclohexane and stored at ~4 °C.

### 2.5. Synthesis of NaGdF_4_:Yb/Er@NaYb_0.9_F4:Nd (10%)@NaYF_4_ Core–Shell–Shell Nanocrystals

At room temperature, 2 mL of the Y(CH_3_CO_2_)_3_ stock solution, 4 mL of oleic acid, and 6 mL of 1-octadecene were added into a 50 mL two-neck flask. The flask was left uncapped, and the resulting mixture was heated to 165 °C for 40 min under a moderate stirring speed to obtain a clear light-yellow solution and then cooled naturally to room temperature under continuous stirring. The reaction mixture was cooled down slowly to room temperature while stirring. Next, 3 mL of the prepared NaGdF4:Yb/Er@NaYbF4:Nd core–shell particles (64.8 mg mL^−1^), 1 mL of NaOH stock solution, and 3.3 mL of NH_4_F stock solution were injected into the reaction flask. The resulting cloudy solution was heated to 50 °C and kept at this temperature for 40 min. The solution was heated to 100 °C for 10 min to remove low-boiling solvent and then heated to 295 °C for 1 h under nitrogen atmosphere. After the reaction was complete, the reaction was stopped and slowly cooled down to room temperature under continuous stirring. Then, the subsequent purification steps are the same as those used for core–shell nanocrystals NaGdF4:Yb/Er@NaYbF4:Nd (10%). Finally, the synthesized NaGdF4:Yb/Er@NaYbF4:Nd(10%)@NaYF4 (122 mg·mL^−1^) core–shell-shell nanocrystals were re-dispersed in 4 mL of fresh cyclohexane and stored at ~4 °C for further use.

### 2.6. Surface Modification of UCNPs-PAA

In a sealed jar, 1 mL of poly(acrylic acid) (PAA, Mw = 1800 Da), 1 mL of UCNPs (15 mg/mL), and 2 mL of ethanol were mixed and stirred overnight. The product was centrifuged with ethanol at 6000 r.p.m. for 20 min, and then the supernatant was discarded. The resultant solution was then washed two times with ethanol/water (1:1 *v*/*v*). Finally, the UCNPs—PAA were collected and re-dispersed in 4 mL of DI water.

### 2.7. In Vitro Cytotoxicity Assays

A549 human non-small lung adenocarcinoma cell line and B16-F10 mouse skin cancer cell line, which was used as a model for human melanoma, were obtained from the American Type Culture Collection (ATCC number; CCL-185 and CRL-6475, respectively). Both cells were used between passages 8 and 17, and routinely cultured in Dulbecco’s Modified Eagle’s Medium (DMEM), supplemented with 10% (*v*/*v*) fetal bovine serum (FBS), penicillin 100 U/mL, and streptomycin 100 μg/mL.

The cytotoxicity of the CSS UCNPs-PAA was examined using 3-(4,5-dimethylthiazol-2-yl)-5-(3-carboxymethoxyphenyl)-2-(4-sulfophenyl)-2H-tetrazolium (MTS) reagent and a Lactate Dehydrogenase (LDH) assay kit to determine the metabolic activity and the membrane integrity of treated cells, respectively. MTS reagent (CellTiter 96^®^ AQueous One Solution Cell Proliferation Assay) was purchased from Promega (Southampton, UK), and the LDH assay kit (In Vitro Toxicology Assay Kit) was supplied by Sigma-Aldrich (Poole, UK). A549 and B16-F10 cells were seeded into 96-well plates at a seeding density of 1 × 10^4^ cells/well, and were incubated overnight in a humified 5% CO_2_ cell culture incubator at 37 °C. Culture growth medium was removed and replaced with fresh medium that contained the CSS UCNPs-PAA, applied at different concentrations (from 0.39 to 800 µg/mL). Cells incubated with medium only were used as the positive control, whereas cells incubated with 0.2% (*v*/*v*) Triton X-100 were used as the negative control. Cells were incubated with the CSS UCNPs-PAA for 4 h. In case of MTS assay, all tested samples were removed, and 100 μL of medium was then added into each well, followed by adding 20 μL of the MTS reagent. Cells were then incubated for 2–3 h at 37 °C. MTS absorbance was thereafter measured at 490 nm using a Cytation™ 3 absorbance microplate reader (BioTek Instruments Inc., Winooski, VT, USA). Percentage of cell viability was calculated using the following equation:

*Cell viability* (%) = *(S − T)/(H − T) 𝑥* 100

where ***S*** is the absorbance of the cells treated with CSS UCNPs-PAA, T is the absorbance of the cells treated with Triton X-100, and H is the absorbance of the cells treated with DMEM.

In the case of the LDH assay, 50 μL of the tested sample solution containing the medium and the released LDH from the disrupted cell membrane caused by the added samples was removed from each well and placed into a new 96-well plate. An amount of 100 μL LDH reagent was applied to the test samples and incubated for 30 min at room temperature. After this period, absorbance at 490 nm was measured using a Cytation™ 3 absorbance microplate reader. LDH release and the percentage of cell viability were calculated using the following equation:

*LDH release* (%) = *(S−H)/(T− H) 𝑥* 100

*Cell viability* (%) = 100 − *LDH release* (%)

### 2.8. Optical Characterization Setup

To analyze the optical properties of the CSS UCNPs, we designed and built a confocal laser-scanning microscope. This confocal microscope was equipped with high magnification microscope objective (100×) and multicolor lasers. A thin layer of the CSS UCNPs was scanned in x–y directions by NIR lasers (808 and 980 nm) (max power = 250 mW) using Galvano (10 mm mirrors) scanners. The fluorescence spectra were collected through the same microscope objective and analyzed with a custom-made spectrometer equipped with a starlight camera (Trius camera model SX-674), and a photon counter (Hamamatsu photon counter model number H7155-21).

## 3. Results and Discussion

**Figure 1:**
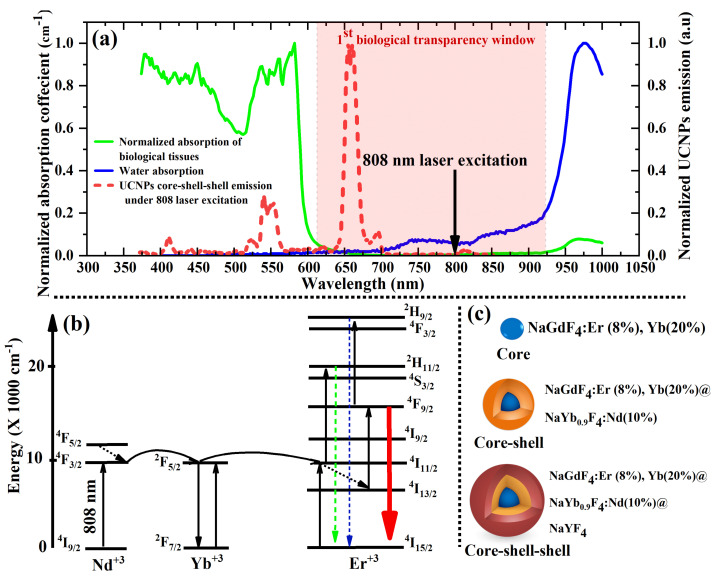
(**a**) Illustration of a superimposed photoluminescence of red enhanced emission of the synthesized CSS UCNPs under 808 nm continuous laser excitation, the illumination and absorption spectra of distilled water (DI water), and biological tissue taken over the visible and the near-infrared (NIR) range. (Data presented in Figure 1a were experimentally collected for this present study). (**b**) Electronics structure and energy transfer processes among Nd^+3^, Yb^+3^, and Er^+3^ of red enhanced CSS UCNPs (photon upconversion) under 808 nm laser excitation. (**c**) An overview of the synthesized UCNP architectures and doping ratio at each stage.

Experimentally, we synthesized bright UCNPs in a CSS structure within the optical transparency window, where tissue scattering and absorption are minimum and photo-toxicity is greatly reduced, as illustrated in Figure 1a. A Neodymium (Nd^+3^) sensitizer was introduced to CSS UCNPs to shift the most common UCNP sensitizer (Yb^+3^) to a biocompatible excitation wavelength (800 nm), as shown in Figure 1b. The C, CS, and CCS UCNPs presented in this work were synthesized following a solvent thermal protocol previously reported in [31] and detailed in the material and methods section. For this, as illustrated in Figure 1c, we first synthesized the core particles NaGdF_4_:Yb (20%) and Er (8%) doped with a relatively high concentration of 8% upconverting erbium (Er^+3^) ion; this doping ratio was reported to give the optimum red enhanced emission of UCNPs [22,23] at low irradiation intensity. To enhance the brightness of the UCNPs, a second Yb^+3^ enriched shell was added to maximize NIR absorption from a 980 nm laser; the second shell also contained another sensitizer Nd^+3^ that absorbs at biocompatible 808 nm irradiation, where laser-induced local overheating effects in living cells are minimized. It should be noted that the Nd^+3^ concentration in the second shell was carefully chosen to be 10% to avoid deleterious energy transfer with Er^+3^ ions in the core particles [32,33]. Finally, we added an inert shell NaYF_4_ to prevent the resonant energy transfer from the UCNP synthesizer (Yb^+3^) to surface defects and vibrational modes of surrounding solvents [22,23].

**Figure 2:**
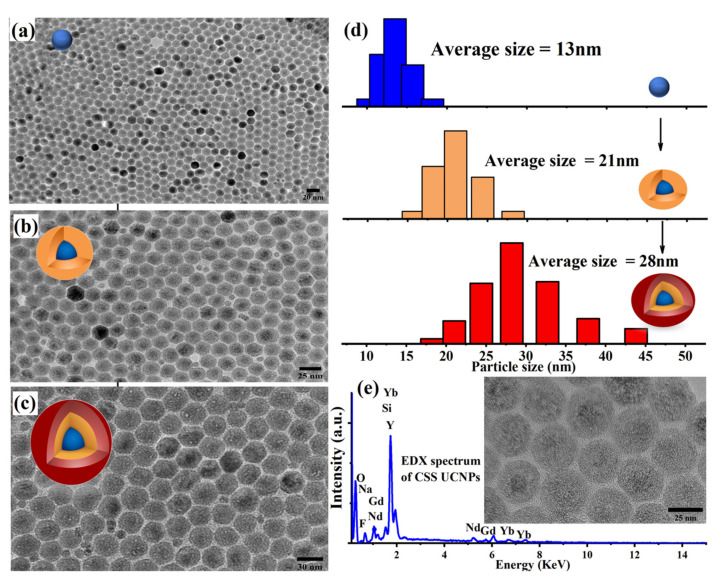
(**a**–**c**) Structural characterization of the core–shell–shell NaGdF_4_:Yb (20%), Er (8%)@ NaGdF_4_:Yb (20%), and Er (8%). (a) TEM images of the C particles NaGdF_4_:Yb (20%), Er (8%). (**b**) TEM images of the CS particles NaGdF_4_:Yb (20%), Er (8%)@ NaYb_0.90_F_4_:Yb (10%), and Er (10%). (**c**) TEM images of the CSS particles NaGdF_4_:Yb (20%), Er (8%)@ NaYb_0.90_F_4_:Yb (10%), and Er (10%)@ NaYF4. (**d**) Corresponding DLS size distributions of the core–shell–shell UCNPs at different synthesis stages. (e, inset) A high resolution TEM image of the CSS UCNPs. (**e**) EDX spectrum recorded from the synthesized CSS UCNPs.

The shape and size of the synthesized CSS particles were characterized using a transmission electron microscope (TEM) and dynamic light scattering (DLS). For TEM imaging, three samples of core (C), core–shell (CS), and then CSS UCNPs were prepared by mixing 1 µL of each sample batch with 90 µL of cyclohexane and dropped on carbon TEM grids. TEM images show dispersed and well crystalline C, CS, and CSS UCNP particles with average sizes of 13–15 nm, 21 nm, and 28 nm, respectively, as demonstrated in Figure 2a–c. The size of synthesized UCNPs was further confirmed by DLS, as shown in Figure 2d. The inset of Figure 2e shows a high resolution TEM image that shows a pure hexagonal crystal lattice of the CSS UCNPs. The energy-dispersive X-ray (EDX) spectrum confirmed the exact composition of the synthesized CSS UCNPs, as illustrated in Figure 2e.

**Figure 3:**
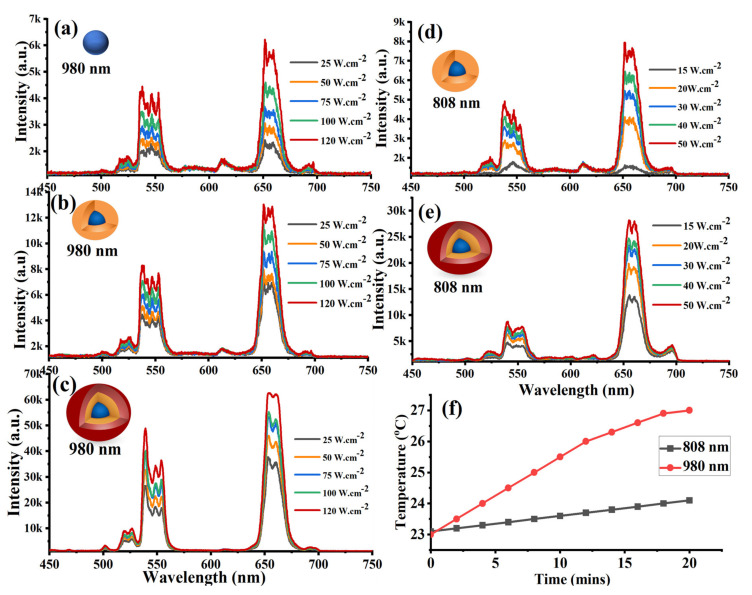
Upconversion luminescence spectra (UCL) of (**a**) core particles NaGdF_4_:Yb (20%) and Er(8%). (**b**) Core–shell particles NaGdF_4_:Yb (20%) and Er (8%)@ NaYb_0.90_F_4_:Nd (10%). (**c**) Core–shell–shell particles NaGdF_4_:Yb (20%) and Er (8%)@ NaYb_0.90_F_4_:Nd (10%) @ NaYF_4_ UCNP particles under 980 nm laser excitation at different laser intensities. UCL of (**d**) core–shell particles NaGdF_4_:Yb (20%) and Er (8%)@ NaYb_0.90_F_4_:Nd (10%). (**e**) Core–shell–shell particles NaGdF_4_:Yb (20%) and Er (8%)@NaYb_0.90_F_4_:Nd (10%)@ NaYF_4_ UCNP particles under 808 nm laser excitation at different laser intensities. (**f**) Comparison of temperature rise of 1 mg/mL CSS-PAA UCNPs dispersed in 2 mL of distilled water under 808 and 980 nm laser irradiation at 50 W ·cm^−2^ for 20 min.

The optical properties of the synthesized C, CS, and CCS UCNPs were studied under 808 and 980 nm lasers. A thin layer of each sample was spin-coated on a piece of quartz to avoid undesired agglomerated emitters and then studied using a custom-made confocal laser-scanning microscope equipped with two laser diodes (808 and 980 nm) (see optical characterization setup). Figure 3a–c shows that under 980 nm laser excitation, the C, CS, and CCS UCNPs illustrated two green emissions at two bands (520 and 550 nm), and also an enhanced red emission at 650 nm. This enhanced emission corresponds to the energy transitions ^2^H_11∕2_ → ^4^I_15∕2_, ^4^S_3∕2_ → ^4^I_15∕2_, and ^4^F_9∕2_ → ^4^I_15∕2_ of the Er^+3^ ion, which is attributed to the optimized concentration of the upconverting (8% Er^+3^) ion in the core nanoparticles [22,23]. This red emission was further enhanced by utilizing a protective shell and optimizing the conventional UCNP sensitizer (Yb^+3^) in the second shell to 92% of the dopant ratio, as seen in Figure 3b,c. This enhancement was about 2.6 times compared to the core nanoparticles under 980 nm irradiation with laser intensity of 50 W·cm^−2^, as illustrated in Figure 3b. In agreement with [23], a remarkable red emission enhancement by a factor of 17× under the same 980 nm laser intensity irradiation was observed after adding the final inert shell to CSS UCNPs. This is because the final inert shell greatly reduced the resonant energy transfer from excited Yb^+3^ ions to the surface defects and to vibrational modes of the surrounding solvent [13,23,24,25,26].

As water is the main ingredient in most biological samples, an excitation wavelength with minimal water absorption is required to overcome the laser-induced local overheating effects in living cells. This is the motivation for adding Nd^+3^ because it has a large absorption band around 800 nm. Briefly, as shown in Figure 1b, the 808 nm laser excites Nd^+3^ in the shell to its ^4^F_5∕2_ excited state, followed by non-radiative relaxation to the ^4^F_3∕2_ state. Energy transfer then populates the ^2^F_5∕2_ state of nearby Yb^+3^, and further energy transfer processes bring the excitation into the core. In the core, the energy transfer from the ^2^F_5∕2_ excited state of Yb^+3^ promotes Er^+3^ to its ^4^I_11∕2_ metastable state. Sequentially, a second e excited state of Yb^+3^ further excites the Er^+3^ to highly excited (^2^H_11∕2_, ^4^S_3∕2_, and ^4^F_9∕2_) Er^+3^ states via multiphonon relaxations. Consequently, two green emissions and a strong red emission occur according to these transitions: ^2^H_11∕2_ → ^4^I_15∕2_, ^4^S_3∕2_ → ^4^I_15∕2_, and ^4^F_9∕2_ → ^4^I_15∕2_, respectively. Figure 3d,e show an excellent UCL with red enhanced intensity from the CS UCNPs at a relatively low 808 nm laser intensity. The addition of the final inert shell enhanced the UCL by 3× times. At 808 nm laser excitation with intensity of 50 W·cm^−2^, the UCL of the CSS UCNPs is comparable to the UCL of the same particles under 980 nm laser excitation.

The quantum yield (Q.Y) of the synthesized C, CS, and CSS UCNPs was measured in this study. It was found that the Q.Y value for NaGdF4:Yb/Er core nanoparticles (average size 13–15 nm), as measured with respect to an infrared dye reference (IR-140), is 0.15 ± 0.02% under 808 nm laser excitation with power intensity (100 W·cm^−2^). Interestingly, the Q.Y value for the CS and CSS UCNPs greatly increased to 4.5 ± 0.04% and 4.9 ± 0.03%, respectively, under the same illumination intensity conditions due to reduced surface quenching. This reported that Q.Y is in good agreement with enhanced Q.Y values of core–shell–shell UCNPs reported in [23]. Q.Y of the synthesized CSS UCNPs, exaptational photostability, and red-enhanced emission within the biological transparency window improved under a biocompatible (low power) excitation wavelength, which makes the CSS UCNP particles a competitor of conventional NPs.

Next, to study the minimization of laser heating in water, and assess the toxicity of the CSS UCNPs in living cells, the particles were transferred to the aqueous phase by exchanging the oleate with poly(acrylic acid) (PAA) ligands following a surface modification procedure discussed in method section [26]. Laser heating minimization in water was investigated using 2 mL of (1 mg/mL) of the surface modified CSS UCNPs-PAA dispersed in deionized water that was placed in a quartz vial (1 cm × 1 cm) and examined on a confocal laser-scanning microscope. Under continuous irradiation of two laser diodes (808 and 980 nm) with laser intensity 50 W·cm^−2^ for 20 min, laser irradiation-dependent temperature rise was recorded. A temperature rise of 4 °C was observed under 980 nm laser illumination for 20 min in contrast to 1.5 °C observed under 808 nm laser irradiation for the same period of time, as shown in Figure 3e. Clearly, the lower excitation wavelength suppressed the laser-induced heating effect by diminishing the absorption by water.

Biotoxicity of the synthesized CSS-PAA UCNPs is first step toward biomedical application. The ideal NPs should fluoresce efficiently while not causing cytotoxic effects. In this study, the cytotoxicity screening of different concentrations of the CSS-PAA UCNPs was assessed in vitro against A549 and B16-F10 cell lines to identify “safe” doses for further studies.

**Figure 4:**
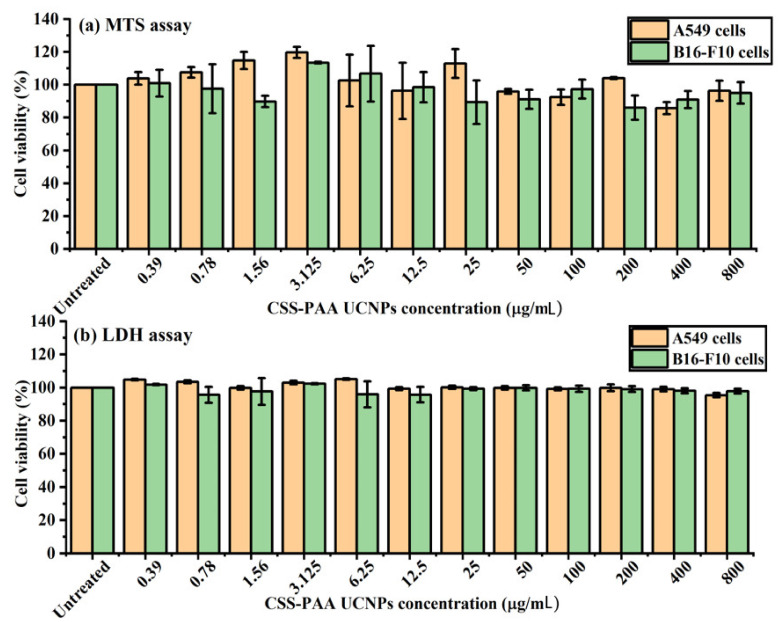
Cell viability of CSS-PAA UCNPs after incubation for 4 h with A549 and B16-F10 cells. These data are the results of (**a**) MTS and (**b**) LDH assays, which are expressed as cell viability (%) and presented as the mean ± SD (n = 3).

The results in Figure 4a showed the effect of different concentrations of the CSS-PAA UCNPs applied to A549 and B16-F10 cells on the metabolic activity examined using MTS assay after 4 h incubation with the cells. Results demonstrated no noticeable effect of the CSS-PAA UCNPs on the cell viability of both cells. High viability of A549 and B16-F10 cells was achieved even at the highest dose applied (800 µg/mL). Basically, there are different reasons that make cell viability higher than 100% in MTS assay. Initially, MTS assay is a relative assay rather than an absolute assay, which can “roughly” indicate whether the applied nanoparticles are harmful to the cells or not by measuring the metabolic activity of the cells. So, the slight increase in cell viability measurements over 100% is acceptable, unless the increase is too far above 100% (not observed in our MTS experiment), which means that the applied nanoparticles may have an influence on cell proliferation. Additional factors could increase the MTS reading above 100%, such as random experimental fluctuation, variation in cell seeding density between the wells, and natural variation in the cellular metabolic activity.

Figure 4b shows the viability of A549 and B16-F10 cells examined using LDH assay after 4 h incubation of the CSS-PAA UCNPs with the cells. Results indicated a similar observation to the MTS assay that high cell viability was obtained at all concentrations applied (from 0.39 to 800 µg/mL). Moreover, the viability level of the lowest and the highest concentrations used was comparable. Comparison of the effect of the CSS-PAA UCNPs on the metabolic viability (determined by MTS assay) and the viability in terms of membrane integrity (determined by LDH release assay) exhibited no change in the viability profile when increasing the dose of applied NPs.

The cellular viability results showed an important corroboration between cell metabolic activity (MTS assay) and LDH release undertaken upon exposure to the CSS-PAA UCNPs. The application of the combination of these assays in this study ensures that the probable toxic effects of NPs are measured from different perspectives—metabolic activity and cell membrane integrity. The in vitro screening of the CSS-PAA UCNPs showed low cytotoxicity effects on A549 and B16-F10 cells, suggesting these NPs as a promising probe for medical applications.

## 4. Conclusions

We synthesized small and bright UCNPs in the CSS architecture. The brightness of the synthesized UCNPs was optimized for NIR absorption in the region of low water absorption, and tuned for red emission via Er^+3^ concentration. Furthermore, two independent toxicity assessments of the synthesized particles were investigated on living cell models to ensure that UCNPs are not toxic for the investigated cell lines, which makes the fluorescent probes promising for bioapplications.

## Data Availability

The data presented in this study are available on request from the corresponding author.
